# Characteristics and outcome of dengue infection; clinical perspective from a secondary care hospital of Karachi

**DOI:** 10.12669/pjms.291.2742

**Published:** 2013

**Authors:** Syed Riazul Hasan, Musarrat Riaz, Farhat Ali Jafri

**Affiliations:** 1 Syed Riazul Hasan, FCPS, Consultant Physician, Remedial Medical Center Hospital, Karachi, Pakistan.; 2 Musarrat Riaz, FCPS, Consultant Physician, Department of Medicine, Baqai Institute of Diabetology and Endocrinology, Baqai Medical University, Karachi, Pakistan. Plot No. 1-2, II-B, Block 2, Nazimabad, Karachi, Pakistan.; 3 Farhat Ali Jafri, MPH, Professor of Community Medicine, Karachi Medical and Dental College, Karachi, Pakistan.

**Keywords:** Dengue fever, Dengue hemorrhagic fever, Dengue shock syndrome

## Abstract

***Objective: ***To determine the frequency and characteristics of dengue fever (DF) in patients of acute febrile illness presenting at a secondary care hospital.

***Methodology: ***The observational cross sectional study was carried out from May to October 2010 in Remedial Centre Karachi and included patients above the age of 12 years who presented with acute febrile illness. The WHO classification and case definitions were used to classify the disease as Dengue Fever (DF), dengue hemorrhagic fever (DHF) and dengue shock syndrome (DSS). Clinical, hematological and biochemical findings were recorded serially until discharge.

***Results: ***During the study period, 90 (34.75%) presented with typical features of DF, 28 (31.11%) were dengue proven, seven (7.7%) proved to be of malaria in which malarial parasites were found positive in the peripheral blood, while the remaining 55 (61.11%) patients were dengue probable. Age of the patients ranged from 13 to 76 years. Fever was the most common clinical presentation (100%) followed by vomiting 50 (55.56%), body ache 31 (34.44%) abdominal pain 17 (18.89%) and headache 9 (10%). Maculopapular rash was seen in 4(4.44%) patients. Laboratory findings included thrombocytopenia, leucopenia and raised alanine aminotransferase levels. Eighty one patients (90%) improved clinically and hematologically and were discharged in stable condition.

***Conclusion: ***Fever and thrombocytopenia were the most common presentation of dengue fever (DF). The overall mortality of DF is low, if treated appropriately. Awareness of health care professionals and public regarding preventive strategies is essential to fight against this disease.

## Introduction

 Dengue fever is a viral illness caused by one of the four serotypes of dengue viruses belonging to the flaiviviridae family.^1^ It has four serotypes DENV1, DENV 2, DENV 3 and DENV 4. All of them are transmitted primarily through mosquitoes of Aedes Egypti. The global prevalence of dengue fever has increased dramatically in the recent decades and the disease is now endemic in many African, Mediterranean region and South East Asian countries.^2^^,^^3^ According to World Health Organization (WHO) more than 2.5 billion people are at risk of dengue infection.^4^ Dengue infection was first documented in Pakistan in 1982 from Punjab^5^ and later the first reported outbreak of dengue haemorrhagic fever in Pakistan was in 1994^6^ and another at upper parts of Punjab in 2003.^7^ Since then it has almost become endemic in various parts of Pakistan with regular epidemic outbreaks especially during the rainy season.^8^

 The spectrum of dengue infection includes dengue fever (DF), a flu like illness with headaches and myalgias, to Dengue Hemorrhagic Fever (DHF) and Dengue Shock Syndrome (DSS), Which is severe and at times fatal disease. DHF is characterized by appearance of hemorrhagic rash or heamorrhagic manifestations in addition to classical DF. DSS is characterized by presence of hypotension and altered mental status.^9^

 Although many studies have been published regarding various aspects of dengue fever in Pakistan^10^^-^^12^ most of them are from tertiary care university hospitals, where patients are generally referred from other health care facilities. The present study was conducted in a secondary care hospital where patients reported early to determine the frequency of dengue fever in patients of acute febrile illness during the studied period.

## Methodology

 This observational cross sectional study was carried out from May to October 2010 in Remedial Centre, a secondary care hospital in Karachi. All patients above the age of 12 years who attended medical outpatients department or were admitted in the hospital due to acute febrile illness were evlaluated for clinical and biochemical features of DF, DHF and DSS. The WHO classification and case definitions were used to classify the disease as DF, DHF and DSS.^13^ Patients with acute febrile illness and clinical features suggestive of dengue with positive serology of dengue specific IgM were labeled as Dengue Confirmed while patients with clinical features suggestive of dengue but negative for dengue serology, malarial parasites and negative blood cultures were considered as dengue probable.

 Clinical hematological and biochemical findings were recorded serially until discharge. Enteric fever was ruled out by performing blood cultures in 26 (28.8%) patients where indicated. Malaria was excluded by peripheral thick and thin film examination and/or immune-chromatographic antigen detection for malaria (ICT) which was done in all patients. Complete blood count and coagulation profile were repeated daily where indicated to monitor the condition of patient. Chest X-Ray and ultrasound of abdomen were performed according to the symptomatology of patients. Symptomatic supportive treatment was advised for all dengue fever patients including intravenous fluids, antiemetics (DImenhydranate) and antipyretics (Acetaminophin). Antimalarial was also given in 7(7.7 %) patients where malarial parasites were positive in the peripheral blood. In cases of haemorrhage, platelet concentrates, packed cells or fresh frozen plasma (FFP) was given where indicated. All the patients were monitored for their clinical, biochemical and hematological profile and the data was recorded.

***Statistical Analysis: ***All variables were entered into statistical package for social sciences (SPSS) version 13.0. Mean and standard deviation was calculated for continuous variables and frequencies for categorical variables. P<0.05 was considered as significant.

## Results

 During the study period 259 patients with febrile illness were seen in the hospital. Out of these, 90 (34.75%) patients presented with typical features of DF and were included in the study. Twenty eight (31.11%) were dengue proven while 55 (61.11%) were dengue probable. Age of patients ranged between 13 to 76 years. There were 30 males while the rest were females. The age range is shown in Table-I.

**Table-I:** Age distribution of patients

*Age(years)*	*n (%)*
13-30	53 (58.89)
31-40	7 (7.78)
41-50	8 (8.89)
>50	22 (24.44)
Total	90

**Table-II:** Clinical manifestation and abnormal laboratory investigations of patients with dengue

* Variables*	*n (%)*
Fever	90 (100)
Body ache	31 (34.44)
Headache	9 (10)
Rash	4 (4.44)
Vomiting	50 (55.56)
Abdominal pain	17 (18.89)
H/O bleeding	1 (1.11)
ALT >35	38 (77.55)
Platelets <50,000	28 (31.11)
WBC<4000	29 (34.12)

 Fever was the most common clinical presentation seen in 100% of patients. Median duration of fever was five days, temperature ranging from (101-105^0^ F) were observed. Other common clinical features were vomiting 50(55.56%), bodyache 31(34.44%), abdominal pain 17 (18.89%) and headache in 09 (10%). A maculopapular rash was seen in 4(4.44%) patients. These clinical features are summarized in Table-II.

Table-III shows the laboratory findings of the patients. The most common hematological abnormality was thrombocytopenia followed by leukopenia. Platelet count below 50,000/cumm was seen in 28 (31.11%). White cell count below 4,000/cumm was observed in 29(34.12%) patients. Prothrombin time (PT) was normal in all patients while alanine aminotransferase (ALT) was elevated in 38(42.2%) patients.

 During the course of illness, platelet count kept on falling and started improving after 4-5^th^ day of admission. Duration of hospital stay ranged from 2-11 days. Eight patients left against medical advice (LAMA) before full recovery while eighty one patients (90%) improved clinically & hematologically and were discharged in stable condition. None of the patient died in our study.

## Discussion

 The present study reports the clinical and biochemical features of DF patients presenting at a secondary care hospital. Among patients who presented with fever, dengue was seen in 28(31.11%) cases. Similar findings are reported in other studies done in Pakistan.^7^^,^^11^

**Table-III:** Outcome of patients

*Outcome*	*n (%)*
Discharge	81 (90)
Referred	1 (1.11)
LAMA	8 (8.89)
Total	90

 Dengue fever is now considered in the differential diagnosis of fever since it was first reported from Karachi in 1994 and another outbreak at upper parts of Punjab in 2003. Thereafter major hospitals of the city reported an increase in the number of cases of dengue probable cases based on clinical and hematological features. Very few cases were proven by dengue serology.

 In our study 59% of patients with DF were below 30 years of age. This is consistent with other studies done locally as well as in other dengue endemic countries like India and Malaysia.^10^^,^^14^^,^^15^ Dengue fever is now considered a major cause of mortality in pediatric population.^15^ Most cases of dengue fever were reported in the month of July, August and September, another finding consistent with other published studies^11^^,^^16^^,^^17^ the reason may be the significant increases in the larval population of mosquito during rainy season which serves as a vector for the transmission of dengue virus among humans. High grade fever with chills and rigors was the main presenting feature and was present in 100% of patients. Typically the onset of fever is sudden associated with severe headache, vomiting, abdominal pain and myalgias. The fever is usually between 101-105 ^0 ^F and lasts for 3-10 days. Rash and hemorrhagic manifestations were seen only in 5 patients unlike some other studies done locally.^10^^-^^12^

 Thrombocytopenia was the most common laboratory manifestation of DF in our patients, a findings consistent with other studies.^11^^,^^18^^,^^19^ Platelet count less than 150X 109/L was seen in 85% of patients in a similar study done in Karachi by Ahmed S et al.^11^ Similarly Mumtaz K et al reported thrombocytopenia in 83% of the study subjects.^10^ The etiology of thrombocytopenia in DF is multifactorial and includes depression of bone marrow, increased destruction of platelets due to infection by virus and presence of antibodies directed against platelets.^20^ Coagulopathy is another commonly reported findings in DF, however in our study none of the patients had prolongation of APTT. Raised liver enzymes were seen in 77% patients, a finding similar with other studies. Marked hepatic dysfunction has been documented in severe cases.^20^^,^^21^ Disseminated intravascular coagulation (DIC) was not seen in any of our patients, a finding which is in contrast to some other studies done locally.^11^^,^^18^^,^^19^

 The overall mortality of dengue infection is low if treated appropriately, however the mortality associated with DHF and DSS is high as these patients need platelets transfusion, ICU settings and ventilator support which is not available in every health care facility. As dengue infection is a recent addition to the already existing endemic infections often reaching to epidemic proportions, the knowledge regarding its presentation, clinical and biochemical features and best management practices are the key to successfully manage these patients. Similarly public awareness regarding preventive strategies is essential to fight against this disease.

## Conclusions

 Fever was the most common clinical presentation and thrombocytopenia laboratory finding observed in patients of DF. The overall mortality of DF is low if treated appropriately. Awareness of health care professional & public regarding preventive strategies is essential to fight against this disease.
